# Asthma in Africa

**DOI:** 10.1371/journal.pmed.0040072

**Published:** 2007-02-27

**Authors:** Matthias Wjst, Daniel Boakye

## Abstract

A new survey shows a recent increase in the prevalence of asthma and allergic diseases in children in Ghana. Wjst and Boakye put this survey into context through a discussion of the epidemiology of asthma in Africa.

*Whatever does not exist does not have a name (African proverb).*

A new survey conducted by Emmanuel Addo-Yobo and colleagues, and published in *PLoS Medicine* [1], shows an increase in the prevalence of asthma and allergic diseases in children in Ghana between 1993 and 2003. In this essay, we discuss the context for this new study by exploring what is known about the epidemiology of asthma in Africa.

## Research on Asthma in Africa

Looking at about 120 papers from Medline on asthma in Africa, we found that asthma research is currently dominated by authors from South Africa, followed by authors from Nigeria, Tanzania, Ethiopia, Kenya, and The Gambia. Most are case studies, closely followed by cross-sectional studies, and then case-control studies. There have been very few cohort studies. Only a few studies use objective measures [2,3], which makes the new *PLoS Medicine* study unusual, since the researchers used repeated measurements of skin prick tests and bronchial reactivity [1].

## Clinical Features and Risk Factors

**Figure 1:**
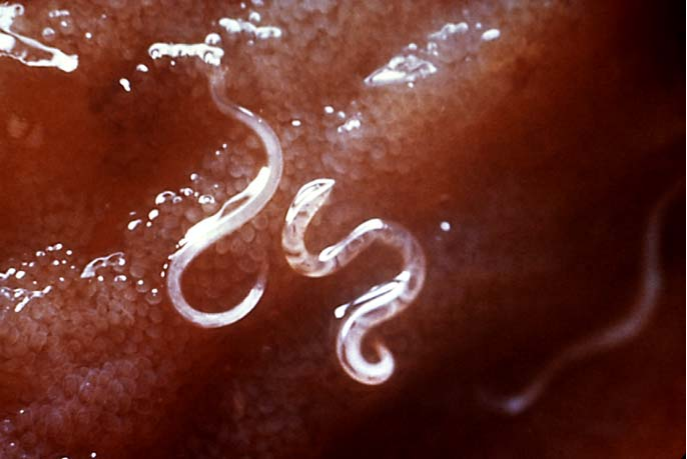
Hookworms *(Ancylostoma caninum)* Attached to the Intestinal Mucosa Barely visible larvae penetrate the skin (often through bare feet), are carried to the lungs, migrate through the respiratory tract to the mouth, are swallowed, and eventually reach the small intestine. This journey takes about a week. (Photo: Centers for Disease Control)

The clinical presentation of asthma in Africa does not seem to be different from other parts of the world [4], although one study reports later disease onset in Africa [5]. Assumed risk factors are local flora such as Kikuyu grass, Makaore cherry, Tanganyika aningré, and Der néré as well as helminthic infection by *Trichuris*, *Schistosoma*, *Ascaris*, and hookworm (Figure 1). Well-known allergens in Africa are house dust mite, cockroach, and cat and dog dander; a less well known allergen is washing soap. Parental history, female sex, low physical activity, and malnutrition, have been described as risk factors together with pesticides, insecticides, wood or kerosene heating, grass mats, mud and cow dung, smoking, and car and truck diesel exhaust. In the occupational setting isocyanate and latex sensitivity have been reported as risk factors, and poultry workers, hairdressers, gold miners, and wood choppers are reported as having an increased risk of asthma. Annual rainfall seems to have an influence on symptom presentation.

Despite the risk factors discussed above, there is no overall hypothesis to explain asthma causation in Africa. In one study, traditional healers in Dar es Salaam, Tanzania were convinced that asthma is caused by “ingestion of amniotic fluid during birth” (83%), by “God” (75%), or “one inherits [asthma] from parents” (73%) [6]. Traditional asthma remedies are usually tried without major success [7] although some may contain pharmacologically active substances [8].

## Prevalence Rates

Intercountry prevalence data are limited to the International Study of Asthma and Allergies in Childhood (ISAAC, http://isaac.auckland.ac.nz) in which seven African countries participated (English-speaking regions: Ethiopia 9.1%, Kenya 15.8%, Nigeria 13.0%, and South Africa 20.3%; and French-speaking regions: Algeria 8.7%, Morocco 10.4%, and Tunisia 11.9% [9]). Symptom rates are lower than in industrialized countries, while only South Africa approaches rates found in the UK. The interpretation of these figures, however, is difficult; there might be an increase with gross domestic product and industrialization factors [10]. Rural African regions always showed much lower asthma prevalence rates than urban areas [11]. People living in rural grasslands rarely, if ever, suffer from allergic diseases and some do not even have a term to describe this condition [12].

## Genes or Environment?

As in industrialized countries asthma in Africa is determined by genes and environment. However, both genetic and environmental effects may operate in different directions and on different scales.

The African environment is different from that in other parts of the world—in Africa, not only climate but also air pollution reaches both extremes [13]. The spectrum of pathogens may also be different, with conflicting evidence on worm infection, IgE, and allergy. Recent research in Ethiopia concludes that “There was no reduction in the risk of AD [atopic dermatitis] in relation to intestinal parasite infection; in fact, AD was increased in subjects with *Trichuris*…. The risk of AD was also unrelated to family size, crowding in the home, or breast-feeding, but was related to previously unrecognized factors including malaria and access to piped drinking water” [14].

Public health experts have long argued that “ingestions of unsafe water, inadequate availability of water for hygiene, and lack of access to sanitation contribute to 1,5 million child deaths” [15]. But how may deaths from unsafe water relate to allergy and asthma? Although it may seem counterintuitive at first, changes in the African gene pool could provide an answer to this question. The concern is not about ethnic differences in asthma-related genes [16–19], although these clearly exist. Gene sequence in Africans is more diverse, showing many more variants [20], but even that may not be a key point. Profound changes in the gene pool may be caused by changes in childhood mortality [21]. At the beginning of the last century acute respiratory infections in Europe had been the main reason for childhood death, causing many children to die before reaching reproductive age. Vaccination programs, better nutrition, and antibiotic treatment, however, have reduced mortality from acute respiratory infection while the asthma incidence increased at the same time. Safe water [14] and antibiotic use [22] could therefore be indicators of a suppression of natural or purifying selection.

This suppression may have enormous consequences on a population level: In Europe, mortality under the age of 5 years was about 250/1,000 live-born children in 1900, dropped to 50 around 1950, and is now about five [21]. In sub-Saharan Africa in 2000, there are still 175 deaths per 1,000 live births [15]; Addo-Yobo et al. report a decline from 69/1,000 to 55/1,000 live births during the study period [1]. Can these differences in childhood mortality explain the extremes of virtually no asthma up to a prevalence of 20%? This seems a testable hypothesis where even the rise or fall of gene variants in immune defence genes could be monitored. Or do environmental factors play a central role? Or is this increase a complex melange of all of the variables?

As of the 1980s, there was an overall conviction that asthma had an anthropogenic origin with indoor and outdoor air pollution as the main culprits. Following some overinterpreted epidemiological findings of the “hygienic” phase, there is now evidence accumulating that the asthma epidemic might have an iatrogenic origin [23]. There might not only be indirect effects of improved living standards and better medical care, there are even direct effects under discussion, for example by oestrogens [24], vitamin D [25], antibiotics [22], and paracetamol [26]. Infant formula (which contains vitamin D) has already entered the food chain in Africa [5]; paracetamol is the most common drug bought over the counter in Ghana (E. O. D. Addo-Yobo, personal communication, 2006). Do African countries offer any unique observations where singular effects of these drugs can be delineated?

## Conclusion

Given the large African continent, its enormous health problems, and its poverty, there needs to be a shift in donor countries' funding priorities [27]. We believe that this shift might be even in the interest of the developed countries that are now suffering from high rates of asthma [9], since major characteristics of traditional lifestyle have changed irrespective of socioeconomic status. Many African societies are still going through the early stages of the transition to urbanised economies, leaving us all opportunities to look for the factors driving early sensitisation and later asthma [27]. In that respect Africa probably has more to offer than any study in the developed world.

## Supporting Information

Alternative Language Text S1 Translation of article into German by Matthias Wjst (102 KB DOC).

## References

[pmed-0040072-b001] Addo-Yobo EOD, Woodcock A, Allotey A, Baffoe-Bonnie B, Strachan D (2007). Exercise-induced bronchospasm and atopy in Ghana: Two surveys ten years apart. PLoS Med.

[pmed-0040072-b002] Keeley DJ, Neill P, Gallivan S (1991). Comparison of the prevalence of reversible airways obstruction in rural and urban Zimbabwean children. Thorax.

[pmed-0040072-b003] Perzanowski MS, Ng'ang'a LW, Carter MC, Odhiambo J, Ngari P (2002). Atopy, asthma, and antibodies to *Ascaris* among rural and urban children in Kenya. J Pediatr.

[pmed-0040072-b004] Sofowora EO (1970). Bronchial asthma in the tropics. A study of 250 Nigerian patients. East Afr Med J.

[pmed-0040072-b005] Yemaneberhan H, Bekele Z, Venn A, Lewis S, Parry E (1997). Prevalence of wheeze and asthma and relation to atopy in urban and rural Ethiopia. Lancet.

[pmed-0040072-b006] Teklu B (1989). Bronchial asthma at high altitude: A clinical and laboratory study in Addis Ababa. Thorax.

[pmed-0040072-b007] Semali IA, Masawe AE (1985). Bronchial asthma as known by traditional healers. East Afr Med J.

[pmed-0040072-b008] Amusan OO, Dlamini PS, Msonthi JD, Makhubu LP (2002). Some herbal remedies from Manzini region of Swaziland. J Ethnopharmacol.

[pmed-0040072-b009] Asher MI, Montefort S, Bjorksten B, Lai CK, Strachan DP (2006). Worldwide time trends in the prevalence of symptoms of asthma, allergic rhinoconjunctivitis, and eczema in childhood: ISAAC Phases One and Three repeat multicountry cross-sectional surveys. Lancet.

[pmed-0040072-b010] Stewart AW, Mitchell EA, Pearce N, Strachan DP, Weilandon SK (2001). The relationship of per capita gross national product to the prevalence of symptoms of asthma and other atopic diseases in children (ISAAC). Int J Epidemiol.

[pmed-0040072-b011] Odhiambo JA, Ng'ang'a LW, Mungai MW, Gicheha CM, Nyamwaya JK (1998). Urban-rural differences in questionnaire-derived markers of asthma in Kenyan school children. Eur Respir J.

[pmed-0040072-b012] Potter PC, Davis G, Manjra A, Luyt D (1996). House dust mite allergy in Southern Africa—Historical perspective and current status. Clin Exp Allergy.

[pmed-0040072-b013] Venn A, Yemaneberhan H, Lewis S, Parry E, Britton J (2005). Proximity of the home to roads and the risk of wheeze in an Ethiopian population. Occup Environ Med.

[pmed-0040072-b014] Haileamlak A, Dagoye D, Williams H, Venn AJ, Hubbard R (2005). Early life risk factors for atopic dermatitis in Ethiopian children. J Allergy Clin Immunol.

[pmed-0040072-b015] Black RE, Morris SS, Bryce J (2003). Where and why are 10 million children dying every year?. Lancet.

[pmed-0040072-b016] Gaillard MC, Zwi S, Nogueira CM, Ludewick H, Feldman C (1994). Ethnic differences in the occurrence of the M1(ala213) haplotype of alpha-1-antitrypsin in asthmatic and non-asthmatic black and white South Africans. Clin Genet.

[pmed-0040072-b017] Awotedu AA, Adelaja AB (1990). Alpha-1-antitrypsin levels and prevalence of Pi variant phenotypes in adult Nigerian asthmatics. Afr J Med Med Sci.

[pmed-0040072-b018] Green SL, Gaillard MC, Song E, Dewar JB, Halkas A (1998). Polymorphisms of the beta chain of the high-affinity immunoglobulin E receptor (Fcepsilon RI-beta) in South African black and white asthmatic and nonasthmatic individuals. Am J Respir Crit Care Med.

[pmed-0040072-b019] Zhou G, Zhai Y, Dong X, Zhang X, He F (2004). Haplotype structure and evidence for positive selection at the human IL13 locus. Mol Biol Evol.

[pmed-0040072-b020] Voight BF, Kudaravalli S, Wen X, Pritchard JK (2006). A map of recent positive selection in the human genome. PLoS Biol.

[pmed-0040072-b021] Wjst M (2004). Is the increase in allergic asthma associated with an inborn Th1 maturation or with an environmental Th1 trigger defect?. Allergy.

[pmed-0040072-b022] Farooqi IS, Hopkin JM (1998). Early childhood infection and atopic disorder. Thorax.

[pmed-0040072-b023] Van Bever HP, Shek LP, Lim DL, Lee BW (2005). Viewpoint: Are doctors responsible for the increase in allergic diseases?. Pediatr Allergy Immunol.

[pmed-0040072-b024] Keski-Nisula L, Pekkanen J, Xu B, Putus T, Koskela P (2006). Does the pill make a difference? Previous maternal use of contraceptive pills and allergic diseases among offspring. Allergy.

[pmed-0040072-b025] Hypponen E, Sovio U, Wjst M, Patel S, Pekkanen J (2004). Infant vitamin D supplementation and allergic conditions in adulthood: Northern Finland birth cohort 1966. Ann N Y Acad Sci.

[pmed-0040072-b026] Shaheen SO, Newson RB, Henderson AJ, Headley JE, Stratton FD (2005). Prenatal paracetamol exposure and risk of asthma and elevated immunoglobulin E in childhood. Clin Exp Allergy.

[pmed-0040072-b027] Broder S, Hoffman SL, Hotez PJ (2002). Cures for the Third World's problems: The application of genomics to the diseases plaguing the developing world may have huge medical and economic benefits for those countries and might even prevent armed conflict. EMBO Rep.

