# Supplementary material for: Asthma in Africa
Source: PLoS Med. 2007 Feb 27;4(2):e72. doi: 10.1371/journal.pmed.0040072 (PMC1808099; doi:10.1371/journal.pmed.0040072)
Supplement: Alternative Language Text S1 — (102 KB DOC). [file pmed.0040072.sd001.doc]

ESSAY

Asthma in Afrika

*Matthias Wjst*, Daniel Boakye*

*Kontaktaddresse

*Was es nicht gibt, braucht auch keinen Namen (afrikanisches Sprichwort)*

Eine neue Studie von Emmanuel Addo-Yobo et. al., in PLoS Medicine zeigt einen Anstieg von Asthma und Allergien bei Kindern zwischen 1993 und 2004 [1]. In dieser Übersicht stellen wir diese neuen Daten in Zusammenhang bisheriger epidemiologischer Forschung in Afrika.

**Forschung zu Asthma in Afrika**

Von den bisherigen 120 Artikel kommen die meisten aus Südafrika, gefolgt von Studien in Nigeria, Tansania, Äthiopien, Kenia und Gambia. Die meisten Studien sind einfache Fallbeschreibungen, viele davon Querschnittsstudien, wenige Fall-Kontroll-Studien und noch weniger Kohortenstudien. Nur wenige Studien beinhalteten objektive Messungen [1], [2]; was die neue *PLoS Medicine* Studie besonders wertvoll macht, sind wiederholte Messungen des Haut-Pricktests und der bronchialer Hyperreaktivität [3].

**Klinische Kennzeichen und Risikofaktoren**

Die klinischen Symptome sind auch in Afrika nicht anders als in anderen Teilen der Welt [4] - lediglich eine Studie berichtete von einem etwas späteren Krankheitsbeginn [5]. Risikofaktoren sind in der lokalen Flora und Fauna zu suchen, zum Beispiel Kikuyu Gras, der Makaore Kirsche oder Holzstaub von Tanganyika aningré und Der néré. Weitere angenommene Risikofaktoren sind Wurminfektionen durch *Trichiuren*, *Schistosomien*, *Askariden* oder *Ankylostomen*. Bekannte Allergene in Afrika sind Hausstaubmilbe, Kakerlake, Katze- und Hundeepithel, weniger bekannt ist Waschseife. Allergie in der Familienanamnese, weibliches Geschlecht und niedrige körperliche Aktivität zusammen mit Unterernährung wurden bisher als Risikofaktoren beschrieben; dazu kommen Pestizide, Insektizide, Holz- oder Kerosinheizung, Matratzen aus Gras, Dreck und Kuhdung, Rauchen, PKW- und LKW-Abgase. Als Berufskrankheiten wurden Isozyanat- und Latexsensibilisierung beschrieben ebenso Erkrankungen im Zusammenhang mit Hühnerhaltung, im Friseurgewerbe, in Goldminen und bei Holzfällern. Die Regenzeit scheint einen Einfluss auf die Symptomatik zu haben.

Trotz der Kenntnis vieler auslösender Faktoren gibt es auch in Afrika keine stimmige Hypothese, welche die eigentliche Ursachen erklären würde. Traditionelle Medizinmänner in Dar es Salaam (Tansania) sind davon überzeugt, dass Asthma durch die “Aspiration von Amnionflüssigkeit bei Geburt” hervorgerufen wird (83%), von “Gott gesandt“ ist (75%) oder dass „man es von den Eltern erbt” (73%) [4]. Traditionelle Asthmaheilmittel werden meist ohne großen Erfolg angewandt [6], obwohl manche pharmakologisch aktive Substanzen enthalten [7].

**Häufigkeitsangaben**

Vergleichbare Häufigkeitsangaben sind weitgehend limitiert auf die international ISSAC Studie (isaac.auckland.ac.nz) bei der sieben afrikanische Länder wiederholt teilnahmen (Englisch sprechende Regionen: Äthiopien 9.1%, Kenia 15.8%, Nigeria 13.0% und Südafrika 20.3%; frankophone Länder Algerien 8.7%, Marokko 10.4% und Tunesien 11.9% [8]). Die Symptomraten sind durchweg niedriger, als in industrialisierten Ländern; allenfalls Südafrika kommt an die englischen Häufigkeitsrekorde heran. Die Interpretation fällt jedoch schwer; es scheint einen Zusammenhang mit Bruttosozialprodukt und dem Industrialisierungsgrad zu geben [9]. In ländlichen Regionen Afrikas finden sich durchweg niedrigere Raten von Asthma [10], die Bevölkerung in den Grassteppen Afrikas leidet selten, wenn überhaupt an Allergien, so dass manche Stämme nicht einmal eine Bezeichnung für diese Symptome haben [11].

**Gene oder Umgebung?**

Wie in Industrieländern auch wird Asthma in Afrika durch Gene und Umgebung bestimmt. Genetische und Umgebungseinflüsse können dabei unterschiedlich zusammengesetzt sein, in unterschiedliche Richtung zielen und unterschiedlich stark sein. Afrikanische Umgebungsfaktoren sind zumindest sehr heterogen im Vergleich zu anderen Teilen der Welt— nicht nur Klima, auch Luftverschmutzung erreichen absolute Extremwerte [12]. Das Spektrum von Allergenen ist ebenfalls unterschiedlich, wobei die Angaben zu Wurminfektionen, IgE-Antikörper und Allergien widersprüchlich sind. Eine neuere Studie aus Äthiopien findet „kein vermindertes Risiko für atopische Dermatitis durch Darmparasiten; im Gegenteil, die atopische Dermatitis war häufiger bei Patienten mit Trichiuris … Das Risiko für atopische Dermatitis war unabhängig von Familiengröße, Personen zu Hause oder Stillgewohnheiten – hingegen beeinflusst von bisher nicht bekannten Faktoren wie Malaria oder Zugang zu Trinkwasser“ [13].

Gesundheitsexperten argumentieren schon lange, dass “Trinken von kontaminiertem Wasser, die mangelhaften Versorgung mit Wasser zum Waschen und fehlende sanitäre Einrichtungen für 1,5 Millionen Todesfälle von Kindern verantwortlich sind” [14]. Gibt es auch Todesfälle, die darüber hinaus mit Allergien oder Asthma zusammenhängen könnten? Obwohl auf den ersten Blick nicht einleuchtend, könnte der afrikanische Genpool dabei eine Rolle spielen. Dabei ist sind vermutlich nicht so sehr die unterschiedlichen Allelfrequenzen der mit Asthma assoziierten Gene relevant [15], [16], [17], [18] – obwohl diese eindeutig bestehen. Gensequenzen von Afrikanern sind generell heterogener [19], aber selbst dies dürfte nicht der wichtigste Punkt sein.

Weitreichende Änderungen des Genpools hingegen könnten durch Sinken der Kindersterblichkeit bewirkt sein [20]. Zu Beginn des letzten Jahrhunderts waren Atemwegsinfekte in Europa noch die Haupttodesursache: Viele Kinder starben noch vor Erreichen des fortpflanzungsfähigen Alters. Impfprogramme, bessere Ernährung und Antibiotika reduzierten die Mortalität, die Asthmaraten hingegen stiegen kontinuierlich an. Die Verfügbarkeit von Trinkwasser [13] oder Antibiotika [21] könnten daher auch ein Indikator sein für eine unterdrückte „natürliche“ Selektion.

Der medizinische und soziale Fortschritt hatte jedenfalls enorme Auswirkungen auf Bevölkerungsebene: In Europa lag 1900 die kindliche Mortalität (bis zum Alter von 5 Lebensjahren) noch bei 250 je 1000 Lebendgeborene, fiel auf 50 um 1950 und liegt jetzt bei etwa 5 Todesfällen [20]. In Afrika südlich der Sahara lag die kindliche Mortalität 2000 immer noch bei 175 Todesfällen je 1000 Lebendgeborenen [14]; Addo-Yobo et al. berichten einen Rückgang von 69 auf 55 je 1000 Lebendgeborenen während des Studienzeitraumes [3]. Können also Unterschiede in der kindlichen Mortalität die Unterschiede in den Asthmaraten erklären? Das ist zumindest eine testbare Hypothese, die durch Anstieg oder Abfall von Allelfrequenzen in Genen der Immunabwehr erklärt werden könnte. Oder sind doch weitere Umgebungsfaktoren relevant? Oder ist dies eine komplexe *Melange* all dieser Faktoren?

Mit Ende der 80er Jahre herrschte die Meinung vor, dass Asthma einen anthropogenen Ursprung hat mit Innenraum- aber auch allgemeine Luftverschmutzung als Hauptschuldigen. Nach einigen überinterpretierten Beobachtungen der Hygienehypothese verdichtet sich nun die Evidenz, dass die Asthmaepidemie auch einen iatrogenen Ursprung haben könnte [22]. Damit sind nicht nur die indirekten Effekte eines höheren Lebensstandards und einer besserer medizinischer Versorgung gemeint – es sind auch einzelne Substanzen in Verdacht, u. a. Östrogene [23], Vitamin D [24], Antibiotika [21] und Parazetamol [25]. Säuglingsnahrung (mit Vitamin D Supplementierung) hat längst auch den Markt auch in Afrika erreicht [5]; Paracetamol wird häufig als Selbstmedikation in der Behandlung der Malaria verwandt (Addo-Yobo, pers. Mitteilung, 2006). Bieten die Länder Afrikas die Chance, isolierte Effekte dieser Substanzen zu beobachten?

**Schlussfolgerungen**

In Anbetracht des riesigen afrikanischen Kontinents, seiner großen Gesundheitsprobleme und seiner Armut, brauchen wir ein Umdenken in den Forschungsprioritäten der Geberländer [26]. Wir glauben, dass dieses Umdenken auch im Interesse der Industrieländer liegen könnte, die vor allem von den hohen Asthmaraten betroffen sind [8]. Große Umbrüche im traditionellen afrikanischen Lebensstil zeichnen sich bereits ab, unabhängig und über alle Gesellschaftsschichten hinweg. Viele afrikanische Länder befinden sich noch in der frühen Phase im Übergang zu städtischen Wirtschaftsformen, was uns die Chance gibt, die Faktoren der Sensibilisierung und des späteren Asthma zu verfolgen [26]. In dieser Beziehung können uns Studien in Afrika mehr bieten, als in jedem anderem hoch industrialisierten Land der Erde.

Matthias Wjst

Institut für Epidemiologie

GSF – Forschungszentrum für Umwelt und Gesundheit

Ingolstädter Landstrasse 1

85764 München-Neuherberg, Germany

E-Mail: m@wjst.de

Daniel Boakye

Parasitology Department

Noguchi Memorial Institute for Medical Research

University of Ghana, Accra, Ghana

E-Mail: DBoakye@noguchi.mimcom.net

Danksagung:

Wir danken Dr. John Britton und beiden Reviewern für Ihre Anmerkungen zu einer früheren Manuskriptversion.

Interessenskonflikt:

Wir erklären, dass wir keinen Interessenskonflikt mit Abfassung dieses Manuskriptes haben.

Literatur:

1. Keeley DJ, Neill P, Gallivan S (1991) Comparison of the prevalence of reversible airways obstruction in rural and urban Zimbabwean children. Thorax 46: 549-553.

2. Perzanowski MS, Ng'ang'a LW, Carter MC, Odhiambo J, Ngari P, et al. (2002) Atopy, asthma, and antibodies to Ascaris among rural and urban children in Kenya. J Pediatr 140: 582-588.

3. Addo-Yobo EO, Woodcock, A., Allotey, A., Baffoe-Bonnie, B., Strachan, D., Custovic, A. (2006) Exercise-induced bronchospasm and atopy in Ghana: Two surveys ten years apart. PLOS Medicine #.

4. Sofowora EO (1970) Bronchial asthma in the tropics. A study of 250 Nigerian patients. East Afr Med J 47: 434-439.

5. Yemaneberhan H, Bekele Z, Venn A, Lewis S, Parry E, et al. (1997) Prevalence of wheeze and asthma and relation to atopy in urban and rural Ethiopia. Lancet 350: 85-90.

6. Teklu B (1989) Bronchial asthma at high altitude: a clinical and laboratory study in Addis Ababa. Thorax 44: 586-587.

7. Amusan OO, Dlamini PS, Msonthi JD, Makhubu LP (2002) Some herbal remedies from Manzini region of Swaziland. J Ethnopharmacol 79: 109-112.

8. Asher MI, Montefort S, Bjorksten B, Lai CK, Strachan DP, et al. (2006) Worldwide time trends in the prevalence of symptoms of asthma, allergic rhinoconjunctivitis, and eczema in childhood: ISAAC Phases One and Three repeat multicountry cross-sectional surveys. Lancet 368: 733-743.

9. Stewart AW, Mitchell EA, Pearce N, Strachan DP, Weilandon SK (2001) The relationship of per capita gross national product to the prevalence of symptoms of asthma and other atopic diseases in children (ISAAC). Int J Epidemiol 30: 173-179.

10. Odhiambo JA, Ng'ang'a LW, Mungai MW, Gicheha CM, Nyamwaya JK, et al. (1998) Urban-rural differences in questionnaire-derived markers of asthma in Kenyan school children. Eur Respir J 12: 1105-1112.

11. Potter PC, Davis G, Manjra A, Luyt D (1996) House dust mite allergy in Southern Africa--historical perspective and current status. Clin Exp Allergy 26: 132-137.

12. Venn A, Yemaneberhan H, Lewis S, Parry E, Britton J (2005) Proximity of the home to roads and the risk of wheeze in an Ethiopian population. Occup Environ Med 62: 376-380.

13. Haileamlak A, Dagoye D, Williams H, Venn AJ, Hubbard R, et al. (2005) Early life risk factors for atopic dermatitis in Ethiopian children. J Allergy Clin Immunol 115: 370-376.

14. Black RE, Morris SS, Bryce J (2003) Where and why are 10 million children dying every year? Lancet 361: 2226-2234.

15. Gaillard MC, Zwi S, Nogueira CM, Ludewick H, Feldman C, et al. (1994) Ethnic differences in the occurrence of the M1(ala213) haplotype of alpha-1-antitrypsin in asthmatic and non-asthmatic black and white South Africans. Clin Genet 45: 122-127.

16. Awotedu AA, Adelaja AB (1990) Alpha-1-antitrypsin levels and prevalence of Pi variant phenotypes in adult Nigerian asthmatics. Afr J Med Med Sci 19: 285-290.

17. Green SL, Gaillard MC, Song E, Dewar JB, Halkas A (1998) Polymorphisms of the beta chain of the high-affinity immunoglobulin E receptor (Fcepsilon RI-beta) in South African black and white asthmatic and nonasthmatic individuals. Am J Respir Crit Care Med 158: 1487-1492.

18. Zhou G, Zhai Y, Dong X, Zhang X, He F, et al. (2004) Haplotype structure and evidence for positive selection at the human IL13 locus. Mol Biol Evol 21: 29-35.

19. Voight BF, Kudaravalli S, Wen X, Pritchard JK (2006) A map of recent positive selection in the human genome. PLoS Biol 4: e72.

20. Wjst M (2004) Is the increase in allergic asthma associated with an inborn Th1 maturation or with an environmental Th1 trigger defect? Allergy 59: 148-150.

21. Farooqi IS, Hopkin JM (1998) Early childhood infection and atopic disorder. Thorax 53: 927-932.

22. Van Bever HP, Shek LP, Lim DL, Lee BW (2005) Viewpoint: are doctors responsible for the increase in allergic diseases? Pediatr Allergy Immunol 16: 464-470.

23. Keski-Nisula L. PJ, Xu B., Putus T., Koskela P. (2006) Does the pill make a difference? Previous maternal use of contraceptive pills and allergic diseases among offspring. Allergy [preprint] doi: 10.1111/j.1398-9995.2006.01201.x.

24. Hypponen E, Sovio U, Wjst M, Patel S, Pekkanen J, et al. (2004) Infant vitamin d supplementation and allergic conditions in adulthood: northern Finland birth cohort 1966. Ann N Y Acad Sci 1037: 84-95.

25. Shaheen SO, Newson RB, Henderson AJ, Headley JE, Stratton FD, et al. (2005) Prenatal paracetamol exposure and risk of asthma and elevated immunoglobulin E in childhood. Clin Exp Allergy 35: 18-25.

26. Broder S, Hoffman SL, Hotez PJ (2002) Cures for the Third World's problems: the application of genomics to the diseases plaguing the developing world may have huge medical and economic benefits for those countries and might even prevent armed conflict. EMBO Rep 3: 806-812.
